# Compact, thermoelectrically cooled surface emitting THz QCLs operating in an HHL housing

**DOI:** 10.1515/nanoph-2025-0185

**Published:** 2025-06-30

**Authors:** Sebastian Gloor, Adrian Weisenhorn, Léo Hetier, Urban Senica, Richard Maulini, Mattias Beck, Jérôme Faist, Giacomo Scalari

**Affiliations:** ETH Zürich, Zürich, Switzerland; Alpes Lasers S.A., Avenue des Paquiers 1, 2072 St-Blaise, Switzerland; Harvard University, Cambridge, MA, USA

**Keywords:** THz QCL, surface emission, thermoelectric operation, compact packaging, loop mirror

## Abstract

Terahertz (THz) technology is experiencing rapid progress thanks to recent advancements in sources and detectors. We present a 3.9 THz quantum cascade laser operating in a compact high heat load (HHL) housing. The device features a loop mirror back reflector and a dry-etched surface-emitting antenna that shapes the far-field pattern to optimize coupling to user-defined devices. A peak power of 1.8 mW and average power of 4.5 μW at 185 K are measured experimentally, and the device output is easily detected with a room-temperature THz camera.

## 1 Introduction

**Figure 1:**
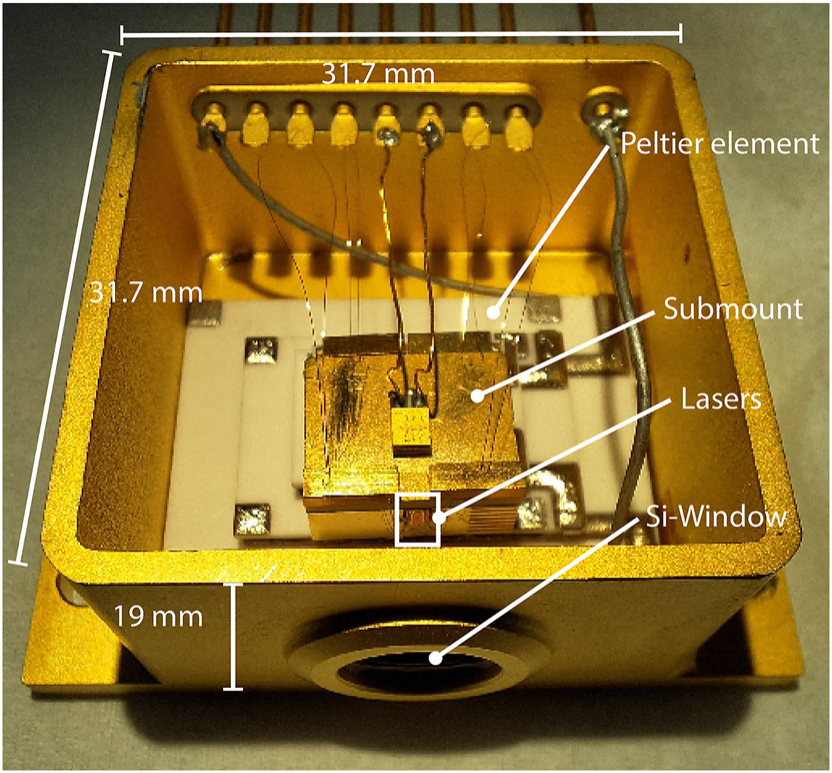
Picture of an unsealed HHL housing measuring 44.5 × 31.7 × 19 mm^3^ with devices mounted vertically resulting in forward emission through a semi-insulating, antireflection coated Si-window. For measurements, the housing could be sealed under vacuum with the Peltier element reaching temperatures down to 185 K.

THz radiation has been proposed for many applications spanning metrology, sensing, security, and high-speed communications [1]. However, a major obstacle for THz applications has been the lack of compact sources with sufficient power output [2]. On the low end of the frequency spectrum, frequency multiplication chains are convenient sources, but their power output drops rapidly above 1 THz [3]. In the range between 1 and 6 THz, photoconductive antennas are commonly used in TDS systems but they are limited in their average output power, still rolling off exponentially above 2 THz [4]. The Quantum Cascade Laser (QCL) is a unipolar, electrically pumped semiconductor heterostructure laser capable of producing high-power coherent THz radiation [5], [6], [7]. The main drawback of THz QCLs is their limited maximum operating temperature, mostly restricting them to cryogenic operation. Recent advances have pushed the maximum operating temperature to 261 K, enabling use on single-stage thermoelectric coolers [8], [9]. To reach the necessary average powers for imaging with a THz camera, the lasers were cooled to 15–40 K below the maximum operating temperature, requiring three-stage thermoelectric coolers. Minimizing losses for highest possible operating temperature requires large device areas and, as a consequence, large operating currents. This places limits on the obtainable duty cycles and average output powers, as the device heats up during operation. At higher duty cycles, it might turn off entirely or overload the cooler’s heat extraction capacity. Active regions for high temperature operation generally maximize carriers available for optical transitions by shortening the module resulting in short transit times and high current densities for a given doping level. From thermal considerations, lower current densities would be beneficial, but while the optimal doping density for THz QCLs is less clear than for MIR QCLs, there is an evident degradation below some critical value, where reduced carrier density will negatively impact population inversion decreasing gain below the propagation losses, mainly due to metals, in the waveguides [10], [11]. Three-well active regions were cooled by five-stage Peltier coolers and achieved duty cycles of 3–4 % at a heatsink temperature of 170 K, 20 K below *T*_max_, and showed an average power output of 100 µW [12]. The thermoelectric cooler used needed large input powers to reach the desired temperatures and was driven with 15A at 32 V. Although the lower dissipation increases the achievable duty cycles, lower *T*_max_ and the conventional ridge geometry hinders the average output power close to the maximum operating temperature of the devices. To achieve higher average output powers close to the cooling floor of thermoelectric coolers, it is advantageous to utilize cavity geometries that show higher thermal conductivity facilitating heat transfer from the active region and lower absolute heat dissipation by the device [13]. An approach using thin and narrow ridges led to considerable improvements in continuous-wave operation in THz QCLs compared to thicker active regions as the out-of-plane thermal conductivity of heterostrucutures is greatly reduced compared to bulk material [14]. The increase in waveguide losses due to the increased overlap of the optical mode with the metal layers makes this approach less viable for increasing average output powers on thermoelectric coolers [15]. THz QCLs almost exclusively employ double metal waveguides as they provide a very favorable product of gain and overlap factor g_th_Γ [16]. A drawback of double metal waveguides is the large impedance mismatch between the confined and free-space optical mode at the facet resulting in low output powers and patterned far fields as was shown in Ref. [17]. Watt-level power output has been demonstrated in single-plasmon THz QCLs, but increased output power and beam quality comes at the expense of lower field confinement reducing maximum operating temperature significantly compared to double metal waveguides [18]. Lenses can be mounted to edge emitting devices to improve beam shape but risk damaging the facets and device failure [19]. Another approach uses a graded photonic structure to force the laser to operate on a radiative mode but it comes with an increase in optical losses [20]. For a monolithic approach without significantly increasing waveguide losses, improved far fields and output coupling in double metal devices can be achieved by planarized passive patch array antennas [21], [22]. However, this introduces a series of additional processing steps increasing the complexity and duration of the device fabrication. In this paper, we present THz QCLs based on an active region lasing up to 210 K, processed into small (1.25 mm × 230 µm and 1.95 mm × 230 µm) footprint devices featuring a high reflectivity looping structure to minimize mirror losses at the back facet and surface-emitting patch array antennas, defined by dry etching its shape directly into the active material waveguide [23]. Device areas were kept small to be pumped at reduced currents between 2.5 A and 3.5 A, compared to 9 A for wide ridge devices on the same active region [24]. Lasing can be observed up to a heatsink temperature of 191 K–197 K. These devices were used for thermoelectrically cooled operation in a high heat load (HHL) housing. This compact hermetic housing with dimensions of 44.5 × 31.7 × 19 mm^3^ is the industry standard for the packaging of mid-infrared QCLs. The lasers chips were mounted on an L-shaped submount, so that the emission is parallel to the base of the package and is outcoupled through a forward facing, semi-insulating Si-window, and soldered onto a 4-stage Peltier element, see Figure 1. 

### 1.1 Device design and fabrication

#### 1.1.1 Cavity design

**Figure 2:**
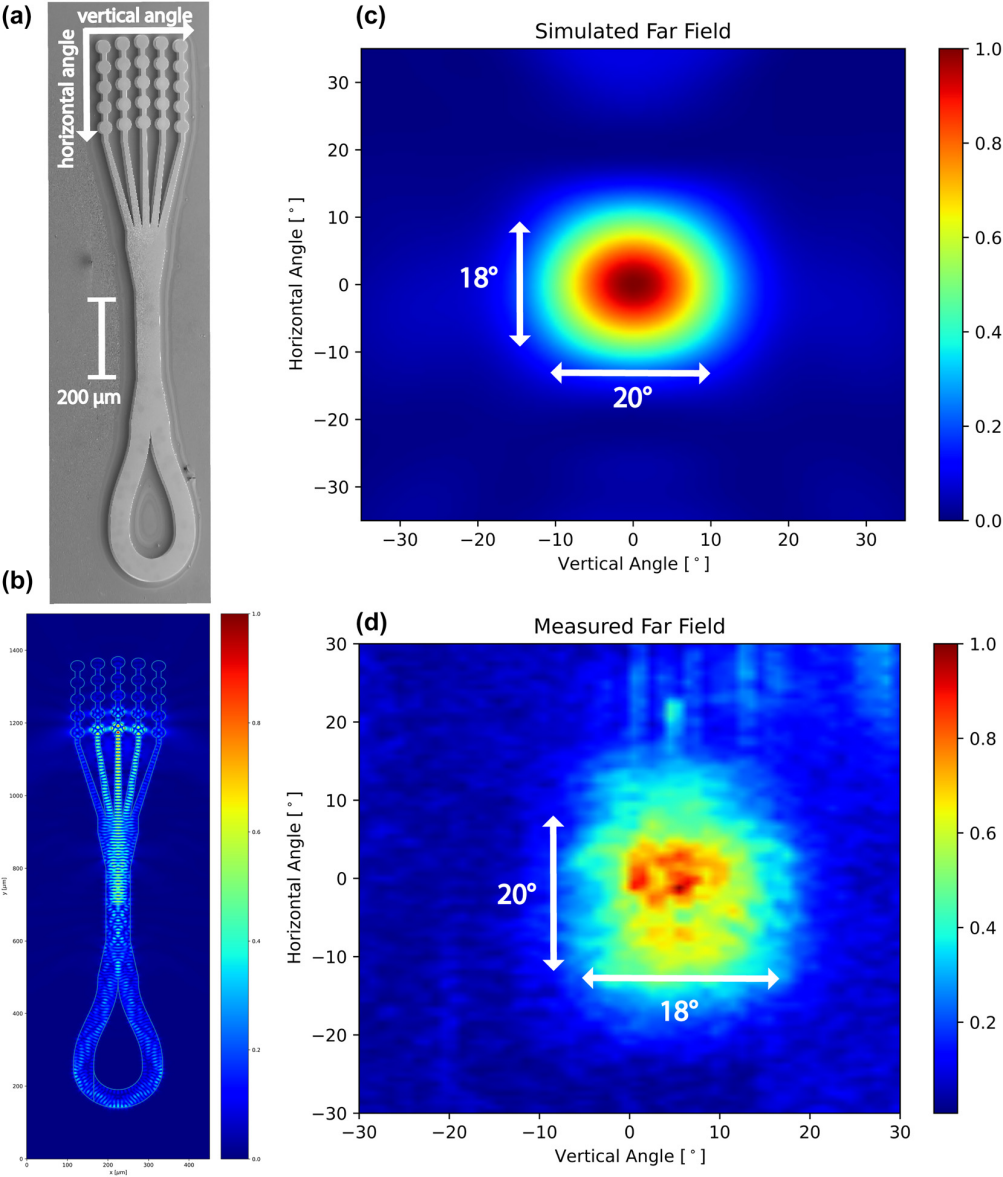
Finite-difference EM simulations for the devices presented. (a) SEM image of a fully processed device. The patch-array antenna results in vertical emission, with the loop mirror reducing back facet mirror losses. (b) Simulation of the electric field inside the full device. From the accumulated phase during a roundtrip, we estimate a free spectral range of 35 GHz. (c) The simulated far field for the dry-etched antenna shows an emission lobe with beam divergence of 18 ° × 20 °. The simulation considers only a single frequency at 3.9 THz in the TM_00_ mode. (d) The experimental far field of an antenna device with loop mirror mounted on a conventional copper mount measured with a pyroelectric detector on a movable stage at a heatsink temperature of 150 K. The vertical shift of the emission lobe is due to the initial alignment of the device. The experimental far field shows a beam divergence of 20° × 18°.

The cavity design for the presented devices was done with both thermal and optical considerations in mind. The devices were kept relatively small to reduce the thermal load on the Peltier element. This places some restrictions on the waveguide length as the antenna consisting of active material is pumped and adds a significant amount of driving current. To achieve surface emission, broadband patch array antennas were developed for planarized THz QCL devices, resulting in high-power surface mission into narrow, single-lobed far-field radiation patterns [22]. To avoid the lengthy and complex planarization that is required for passive antennas, we employ a different approach by directly dry etching a patch array structure into the active region itself. The antenna design was based on the broadband design presented in Ref. [22] but rescaled for the different core material from n_BCB_ ≈ 1.5 to n_GaAs_ ≈ 3.6, with the central emission frequency adjusted to the active region at 3.9 THz. The calculated far field can be seen in Figure 2a and shows a beam divergence of 18° × 20° for the fundamental transversal mode TM_00_. For higher order transversal modes, the antenna shows multilobed emission in the vertical plane. To force the device to operate on the fundamental transversal mode, we tapered the antenna to a straight waveguide of widths of 50 µm–60 µm, which acts as a transversal mode filter by introducing additional losses to higher order modes by increased sidewall roughness scattering and reduced modal overlap with the active region [25]. Transversal mode control generally results in lower *T*_max_ but is necessary to enable vertical emission in a single-lobed far field [26]. Additionally, we designed the cavity to maximize the collected power and minimize the mirror losses. Mirror losses in Fabry–Perot lasers are defined as $\alpha_{m}=-\frac{1}{2L}\log\left(R_{1}R_{2}\right)$ , where *L* is the cavity length and *R*_1_, *R*_2_ are the end mirror reflectivities. The mirror losses are usually moderate due to the large reflectivities provided by cleaved facets (R ≈ 70 % at 3.9 THz [22]), cavity lengths of several millimeters, while the waveguide losses are comparatively large at *α*_wg_ ≈ 20 cm^−1^ (in comparison, *α*_*m*_ ≈ 1.8 cm^−1^ for a 2 mm long cavity with cleaved end facets). We reduce the back facet mirror losses by introducing a loop at the end of the devices that act as a high reflectivity mirror [23]. This result in a reflection between 92 % and 98 % in the span of 2 THz–4 THz and a reduction of total mirror losses by ∼20 % compared to a device with a cleaved back facet. In other works, high reflectivity was achieved by Bragg reflectors, but the loop mirror approach is more broadband, robust, and forgoes the need for planarization entirely [27]. The slope efficiency for a ridge laser can be written as $\eta \propto \frac{\alpha_{m}^{\text{front}}}{\alpha_{m}+\alpha_{\text{wg}}}$ , with *α*_*m*_ the mirror losses and *α*_wg_ the waveguide losses. Using *α*_wg_ = 20 cm^−1^, we expect a 2.9-fold increase in slope efficiency assuming an outcoupling efficiency of 20 % for the antenna as predicted by simulations compared to a 1 mm long ridge with cleaved facets. The absence of a second emission point at the back facet will negate any interference effects that arise in THz devices with two cleaved facets. The cavity length is not clearly defined as our devices do not constitute a standard Fabry–Pérot cavity, but from the phase shift accumulated by the optical field as it traverses the device, we estimate the repetition rate at around *f*_rep_ = 35 GHz, corresponding to a standing wave cavity length of 1.2 mm, which was used for calculations of the mirror losses. A simulation of the electric field of the full device can be seen in Figure 2b. The repetition rate, radiative efficiency, as well as the far field are highly dependent on the refractive index of the mode. The antenna design was done using *n*_ref_ = 3.6. The refractive index controls the emission angle in horizontal direction and a mismatch can result in lower intensity side lobes.

#### 1.1.2 Fabrication and packaging

The devices were fabricated using a new epitaxial growth of the same 2-well active region design as in Ref. [24]. A maximum operating temperature of *T*_max_ = 210 K with a maximum current density of 2.5 kA/cm^2^ was measured for a 120 µm wide, 1 mm long, edge-emitting ridge with cleaved facets. The current density is reduced by 30 % compared to the original growth without a change in maximum operating temperature making this layer ideal for operation on Peltier coolers. The device fabrication followed standard procedure for double-metal THz QCLs, using a SiCl_4_/N_2_ plasma for ICP etching using the top metallization as a self-aligned mask.

The QCL devices were integrated into a compact high-heat-load (HHL) housing designed for optimal thermal and optical performance. Each laser was indium-soldered onto a gold-coated, L-shaped submount machined from copper–tungsten (CuW), which ensures both mechanical robustness and excellent thermal conductivity. This L-shaped geometry enables forward emission through the front-facing high-resistivity float-zone silicon (HRFZ-Si) window of the HHL while maintaining direct thermal contact with a four-stage thermoelectric cooler (SP2394-07AB, Marlow Industries™). The Peltier module is liquid-cooled on the hot side at −15 °C, achieving a temperature differential of up to 85 K in no-load conditions, allowing a minimum operational temperature of 175 K, and up to 185 K when the device is active. The CuW submount acts as a thermal bridge between the QCL and the Peltier cooler, ensuring efficient heat extraction during pulsed operation (with duty cycles between 0.1 % and 1.0 %). After soldering, electrical connections were made by wire bonding the laser and submount to the HHL housing pins. To extract the THz beam, the housing incorporates a semi-insulating HRFZ-Si window, coated on both sides with a custom 13.4 µm thick C-parylene antireflective layer [28]. Finally, a hermetically sealed cover equipped with a vacuum pipe was affixed to the housing, enabling internal evacuation to improve thermal isolation from the ambient environment. The full assembly without the top cover can be seen in Figure 1.

## 2 Experimental results

The devices were first tested on a liquid He flow cryostat with a He-cooled Si-bolometer to determine *T*_max_ and subsequently mounted on a 4-stage Peltier element inside an HHL housing. Two devices with the same length are mounted on a single chip. Two different lengths, 200 µm and 900 µm, for the straight waveguide section were tested. This results in a total device length of 1.25 mm and 1.95 mm for the short and long devices, respectively. Shorter devices showed *T*_max_ of 193 K. This reduction in maximum operating temperature compared to wide ridges originates from increased mirror losses due to the antenna as well as the increased waveguide losses due to sidewall roughness scattering of the narrower waveguides at 50 µm–60 µm. The 1.25 mm long devices double the mirror losses compared to a 1 mm long ridge from *α*_m_ = 3.5 cm^−1^ to *α*_m_ = 6.9 cm^−1^. It has been observed that increasing losses by 5 cm^−1^ can result in reduced *T*_max_ of 20 K, similarly to what we observe in our devices [29]. From mirror loss calculations, we find an estimated ratio for the slope efficiencies of *η*_ridge_/*η*_*antenna*_ = 2.9 agreeing well with the experimentally measured value of ∼3.5. The 1.95 mm long devices achieved a slightly higher *T*_max_ of 196 K where increasing the length further did not result in better temperature performance suggesting limitation by the higher losses from the narrow waveguide section. The far-field pattern of an antenna device with a loop mirror mounted on a conventional copper submount was measured at 150 K with a pyroelectric detector on a movable stage and can be seen in Figure 2d. The far-field pattern shows an emission lobe with 20° × 18° full-width at half-maximum (FWHM) divergence closely matching simulations. The beam properties are close to the passive antennas in Ref. [22], although the rescaling results in larger beamsteering with frequency making this antenna a less broadband approach than planarized variants.

**Figure 3:**
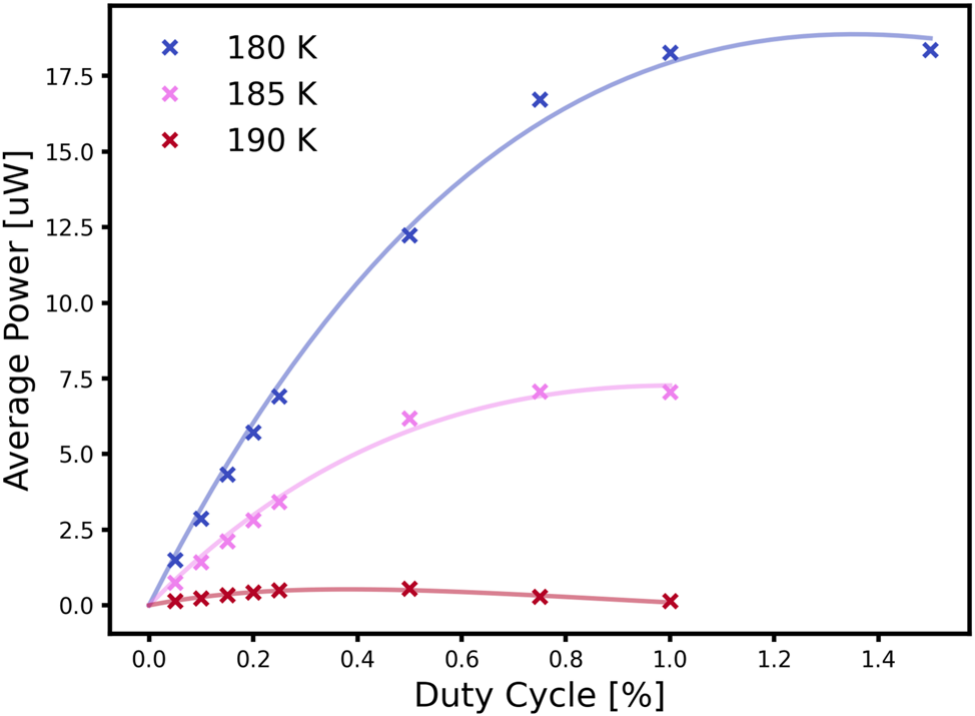
Average power in dependence of duty cycle for small antenna devices mounted on a conventional copper block. Measurements were conducted with a liquid He flow cryostat. The pulse width was fixed at 200 ns, and the duty cycle increased until the signal dropped. Duty cycles for maximum output were in the range 0.5%–0.75 % in a micro–macro pulse setup for an average power of 7 µW. The devices mounted on the L-shaped submount the power output reaches 4.5–2.2 μW.

A duty cycle study was conducted to determine the maximum achievable duty cycle and the average power output of the devices, see Figure 3. Optical powers were deduced by calibrating the Si-bolometer response to a known source. The study was conducted with pulse lengths of 200 ns in a micro–macro pulse scheme. The output power and duty cycle will be lower when the laser is driven in a continuous pulse stream and depends on the maximum heat load that can be extracted by the Peltier element. Shortening the pulse length from 200 ns to 100 ns considerably improves average output powers at a given duty cycle and is taken into account for measurements with the devices mounted in the HHL housing. The shorter devices achieved average output powers of 4.5 µW and 2.2 μW at 185 K with a maximum duty cycle of 0.75 %. For pulse stream measurements, the pulse width was reduced to 100 ns and the duty cyle reduced to 0.25 % to account for the increased macro-pulse duty cycle from 50 % to 100 %. Although the duty cycle was reduced, with the shortened pulse we achieved the same average power output of 4.5 µW.

**Figure 4:**
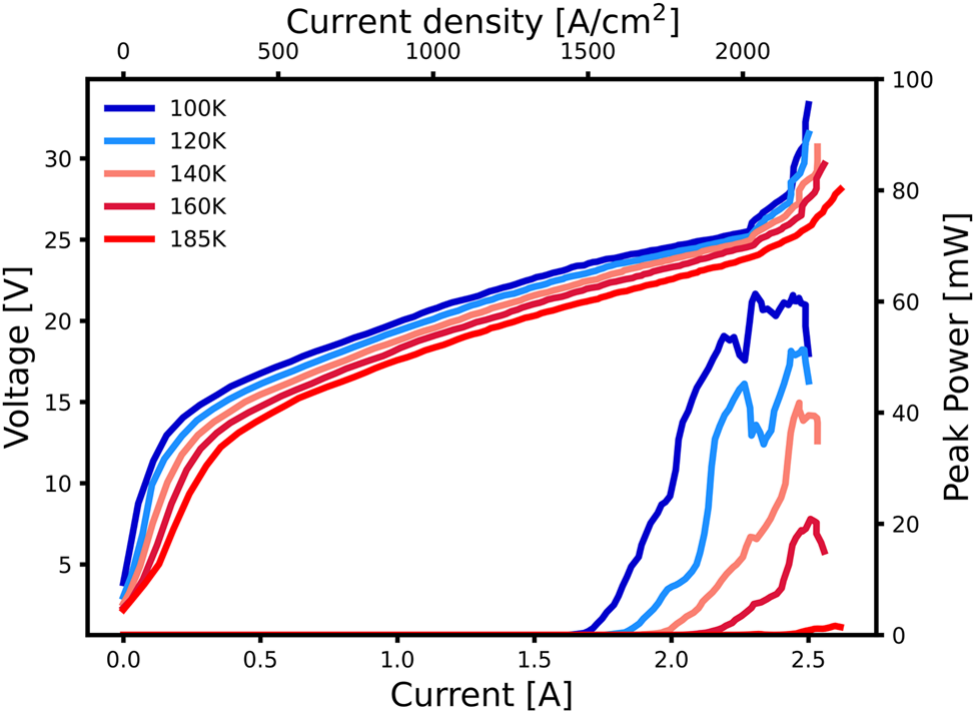
Light–current–voltage measurements of a short antenna device cooled with a liquid nitrogen flow cryostat and Si-bolometer at various temperatures. The measurements were acquired with ≈ 100 ns pulse length at a repetition rate of 415 Hz. The peak power output at 185 K is 1.6 mW.

The average emitted power drops below 1 μW at 190 K. This behavior is expected, as optical power drops very rapidly close to *T*_max_. Longer devices showed average output powers up to 18.5 μW at 185 K and 4 μW at 190 K. The higher *T*_max_ is a major contributing factor for this increased output power but comes at the cost of increased dissipation. While the power output is lower for shorter devices, the electrical dissipation is 30 % lower and, therefore, presents a lower heat load on the Peltier element. The transmission of the AR-coated semi-insulating Si-window was measured with an FTIR and determined to be at 85 %. Power measurements were not adjusted for window transmission. The devices mounted on the L-shaped submount were fully characterized with the Si-bolometer prior to packaging inside the HHL and subsequent spectral and camera measurements. Light–current–voltage measurements for low duty cycle measurements can be seen in Figure 4.

**Figure 5:**
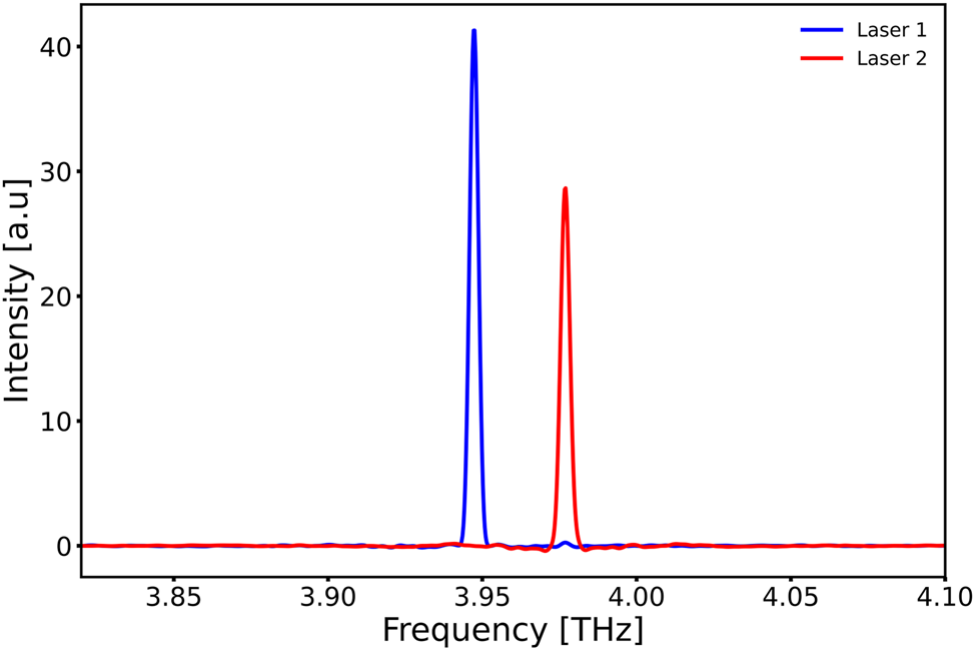
Spectral measurements of two devices with a 200 µm long straight section operating inside the HHL housing. The hot side of the Peltier element was kept at −15 °C and driven with 4.1 A at 6.6 V. Devices were driven with 100 ns long pulses at 0.25 % duty cycle. The interferogram was taken in a single scan over an optical path delay of 12 cm with a room-temperature DTGS detector. From finite-differences simulations, we would expect a repetition rate of 35 GHz where we experimentally measure a mode spacing of 29 GHz.

Spectral measurements of the devices mounted inside the HHL were taken with a commercial Bruker 80v FTIR and a room-temperature DTGS detector. The hot side of the Peltier element was kept at −15 °C with a chiller and the Peltier biased at 6.6 V and 4.1 A. The lasers were driven with 100 ns long pulses at a duty cycle of 0.25 % in a continuous pulse stream. The measured spectra can be seen in Figure 5. The frequency difference between the modes of 29 GHz corresponds with an effective cavity length of 1.41 mm.

**Figure 6:**
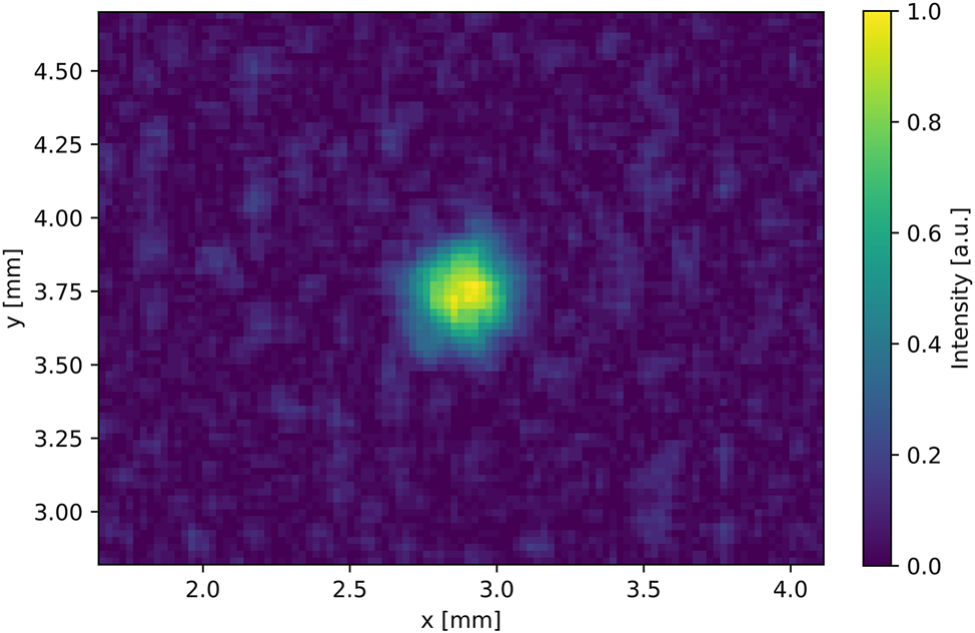
The emission of a THz QCL mounted inside an HHL measured with a THz camera. The beam was focused with two 1.5 inch TPX aspherical lenses at a distance of 15 cm.

To show the useability of these packaged THz QCLs, we imaged the output with a room-temperature THz micro-bolometer camera. While the beam divergence is quite narrow for a THz QCL device, it still has a considerable divergence and its beam waist is larger than the detector area at the measuring distance. The THz output from the laser inside the HHL was focused using two aspheric 1.5 inch TPX lenses with 50 mm focal length. The camera is placed 15 cm in front of the housing without any purging of the optical path. The focused THz spot could easily detected. The measurement of the device with the THz camera is shown in Figure 6.

## 3 Conclusions

We have shown a compact assembly for THz QCLs with average power outputs of 4.5 µW on a thermoelectric cooler with surface-emitting antenna-coupled devices and low-loss loop mirror structures driven at 2.5 A. These results will allow operation outside of specialized labs of existing THz QCL active regions with their maximum operating temperature still below room temperature. To improve the dissipation and thermal resistance, the devices were kept relatively small, but additional structures to improve lateral heat transfer from the active region could be employed [13]. While this would not improve the maximum operating temperature, it would increase the achievable duty cycle and average output power necessary for applications.
